# MicroRNA-338-3p suppresses cell proliferation and induces apoptosis of non-small-cell lung cancer by targeting sphingosine kinase 2

**DOI:** 10.1186/s12935-017-0415-9

**Published:** 2017-04-17

**Authors:** Guowei Zhang, Hao Zheng, Guojun Zhang, Ruirui Cheng, Chunya Lu, Yijie Guo, Guoqiang Zhao

**Affiliations:** 1 Department of Respiratory Medicine, The First Affiliated Hospital of Zhengzhou University, Zhengzhou, 450052 Henan People’s Republic of China; 2 Department of Respiratory Medicine, Henan Cancer Hospital, Affiliated Cancer Hospital of Zhengzhou University, Zhengzhou, 450008 Henan People’s Republic of China; 3 School of Basic Medical Sciences, Zhengzhou University, No.100 Kexue Road, Zhengzhou, 450001 Henan People’s Republic of China; 4 Zhengzhou Foreign Language School, High School (16) Class, Fengyang Road, Zhengzhou, 450001 Henan People’s Republic of China

**Keywords:** MicroRNA-338-3p, Sphingosine kinase 2, Non-small-cell lung carcinoma, Cell proliferation, Apoptosis

## Abstract

**Background:**

Lung cancer is the major cause of cancer-related death worldwide, and 80% patients of lung cancer are non-small-cell lung cancer (NSCLC) cases. MicroRNAs are important gene regulators with critical roles in diverse biological processes, including tumorigenesis. Studies indicate that sphingosine kinase 2 (SphK2) promotes tumor progression in NSCLC, but how this occurs is unclear. Thus, we explored the effect of miR-338-3p targeting SphK2 on proliferation and apoptosis of NSCLC cells.

**Methods:**

Expression of miR-338-3p and SphK2 in NSCLC A549 and H1299 cell lines was measured using qRT-PCR and Western blot. CCK-8 and colony formation assays were used to assess the effect of miR-338-3p on NSCLC cell line proliferation. Flow cytometry was used to study the effect of miR-338-3p on NSCLC apoptosis. Luciferase reporter assay and Western blot were used to confirm targeting of SphK2 by miR-338-3p. Finally, in vivo tumorigenesis studies were used to demonstrate subcutaneous tumor growth.

**Results:**

miR-338-3p expression in 34 NSCLC clinical samples was downregulated and this was correlated with TNM stage. miR-338-3p significantly suppressed proliferation and induced apoptosis of NSCLC A549 and H1299 cells in vitro. SphK2 was a direct target of miR-338-3p. Overexpression of miR-338-3p significantly inhibited SphK2 expression and reduced luciferase reporter activity containing the SphK2 3′-untranslated region (3′-UTR) through the first binding site. SphK2 lacking 3′-UTR restored the effects of miR-338-3p on cell proliferation inhibition. miR-338-3p significantly inhibited tumorigenicity of NSCLC A549 and H1299 cells in a nude mouse xenograft model.

**Conclusions:**

Collectively, miR-338-3p inhibited cell proliferation and induced apoptosis of NSCLC cells by targeting and down-regulating SphK2, and miR-338-3p could inhibit NSCLC cells A549 and H1299 growth in vivo, suggesting a potential mechanism of NSCLC progression. Therapeutically, miR-338-3p may serve as a potential target in the treatment of human lung cancer.

## Background

Lung cancer is the leading cause of cancer related death worldwide. Non-small-cell lung cancer (NSCLC) accounts for 70–80% of lung cancer cases [1, 2]. Recently, advances in clinical and experimental oncology have been made for treating NSCLC [3–6], but its complicated pathology is unclear, and more work is required to identify novel molecules that are involved in the process. Therefore, investigation of the molecular mechanisms underlying NSCLC tumorigenesis may aid in the development of novel therapeutic targets and strategies for the treatment of the malignancy.

MicroRNAs (miRNAs) are small, endogenous, noncoding RNAs of approximately 22 nt that regulate the expression of target mRNA by binding to 3′-untranslated regions (3′-UTRs), resulting in target mRNA degradation or silencing [7, 8]. Recent studies indicate that microRNAs (miRNAs) are important subtypes of noncoding RNAs in the regulation of diverse biological processes, especially those involved in critical pathways linked to cancer cell proliferation, apoptosis, and metastasis [9–11]. One target gene may be regulated by multiple miRNAs and one miRNA may regulate multiple target genes, which results in the formation of complex regulation networks in tumorigenesis [12]. Studies show that miRNAs exert oncogenic or tumor suppressor roles in the etiology and pathogenesis of cancer by targeting tumor suppressors or oncogenes [13, 14].

miR-338-3p is mapped to the seventh intron of the apoptosis-associated tyrosine kinase (*AATK*) gene and miR-338-3p regulates gene AATK expression in rat neurons [15]. miR-338-3p was first reported in prion-induced neurodegeneration: expression of miR-338-3p is reduced in mouse brains infected with mouse-adapted scrape [16]. In tumorigenesis, miR-338-3p is down-regulated in multiple cancers, including gastric, colorectal, and lung cancers [17–19]. However, little is known about the role of miR-338-3p in NSCLC proliferation and apoptosis so we investigated NSCLC progression and development by identifying miRNA targets.

Sphingolipids are a diverse group of water-insoluble molecules including ceramides, sphingoid bases, ceramide phosphates and sphingoid-based phosphates [20], all of which contribute to cell proliferation, invasion and apoptosis [21]. Sphingosine kinases (SphKs) are the rate-limiting enzymes for cellular sphingoid-base phosphates and have two distinct isoforms, SphK1 and SphK2 [22, 23]. SphK1, which is an oncogenic kinase, is involved in tumor development and progression of various human cancers but biological functions of SphK2 in NSCLC remain unknown. Thus, we studied the regulation of miR-338-3p on SphK2 and the consequent effects on proliferation and apoptosis of human NSCLC cells.

## Methods

### Ethics statement

This study was approved by the Ethics Committee of ZhengZhou University (ZhengZhou, China) and full informed consent was provided by all of the patients involved prior to sample collection.

### Patients and tissue samples

A total of 34 patients diagnosed with primary NSCLC at the Henan Tumor Hospital (Zhengzhou, China) between August of 2015 and June of 2016 were included in this study. No patient received chemotherapy or radiotherapy prior to surgery. Tumor and corresponding non-tumor lung tissue samples were collected and rapidly frozen in liquid nitrogen and stored at −80 °C. Tumors were classified according to World Health Organization classification. Data for patient age, gender, smoking history, differentiation, and TNM stage were obtained from patient records (see Table 1).

**Table 1:** Expression of SphK2 and miR-338-3p in tissues of 34 lung adenocarcinoma cases

Parameter	n	Sphk2		miR-338-3p	
		Expression	p value	Expression	p value
Gender					
Male	21	0.539 ± 0.105	0.428	0.330 ± 0.201	0.485
Female	13	0.504 ± 0.148		0.385 ± 0.255	
Age					
<60	16	0.5.34 ± 0.133	0.689	0.304 ± 0.160	0.243
≥60	18	0.517 ± 0.114		0.393 ± 0.260	
Differentiation					
Well	14	0.551 ± 0.120	0.433	0.290 ± 0.121	0.404
Moderate	13	0.491 ± 0.140		0.401 ± 0.239	
Poor	7	0.539 ± 0.104		0.380 ± 0.306	
TNM stage					
I + II	24	0.495 ± 0.108	0.017*	0.406 ± 0.228	0.023*
III	10	0.598 ± 0.127		0.220 ± 0.132	
Smoking history					
Smoker	18	0.523 ± 0.096	0.710	0.299 ± 0.108	0.145
No smoker	16	0.512 ± 0.149		0.410 ± 0.294	

*p < 0.05

### Cell lines and cell culture

Normal human bronchial epithelial cell line NHBE and human lung cancer cell lines H460, H1299, A549, SPC-A-1 and Calu-3 were purchased from the Shanghai Institutes for Biological Sciences, Chinese Academy of Sciences. Cells were cultured in DMEM containing 10% fetal bovine serum (FBS), 100 U/mL penicillin and 100 μg/mL streptomycin at 37 °C in a humidified cell incubator with 5% CO_2_.

### RNA isolation and qRT-PCR

Total RNA was isolated from tissue samples and cell lines using the Qiagen RNeasy kit (Valencia, CA) according to the manufacturer’s instructions. RNA quality and quantity were assessed by standard electrophoretic and spectrophotometric methods. Mature miR-338-3p expression was measured by qRT-PCR according to the Taqman MicroRNA Assays protocol (Applied Biosystems, Carlsbad, CA) and normalized using U6 small nuclear RNA (RNU6B; Applied Biosystems, Carlsbad, CA) with the 2^−ΔΔCt^ method.

### Western blot

Total protein was extracted from tissue samples and cell lines using RIPA buffer containing PMSF. A BCA protein assay kit (Beyotime, Haimen, China) was used to measure total protein. Proteins were electrophoresed via SDS-PAGE and transferred onto PVDF membranes. After blocking, membranes were washed four times with TBST at room temperature and then incubated overnight at 4 °C with diluted primary antibody. Following extensive washing, the membranes were incubated with secondary antibody (HRP-conjugated goat anti-rabbit IgG, 1:3000; Santa Cruz Biotechnology, Santa Cruz, CA). Signals were visualized using a chemiluminescence detection kit (Amersham Pharmacia Biotech, Piscataway, NJ). Antibody against GAPDH (Santa Cruz Biotechnology, Santa Cruz, CA) served as an endogenous reference. Protein intensity was scanned on Typhoon PhosphorImager (GE Healthcare, Pittsburgh, PA) for fluorescent signal. Experiments were performed in triplicate.

### RNA oligoribonucleotides

The miR-338-3p mimics (GMR-miR MicroRNA-338-3p mimics) used in this study were synthesized by Shanghai GenePharma Co. Ltd. Human NSCLC cells A549 and H1299 were seeded into six-well plates (2 × 10^5^ cells/well) and allowed to settle overnight until they were 50–80% confluent. Cells were transfected with miR-338-3p mimics using Lipofectamine™ 2000 (Invitrogen, Carlsbad, CA) according to the manufacturer’s instructions. Three groups were generated for the ensuing experiments: non-transfected group (blank control), scrambled miRNA transfected group (negative control, NC) and miR-338-3p mimics transfected group (inhibitor). Then, 24–48 h after the initial transfection, the cells were harvested for further experiments.

### Plasmid construction and cell transfection

SphK2 coding sequences lacking the 3′-UTR were cloned into the pcDNA3.1 vector (Invitrogen) to generate the pcDNA3.1-SphK2 expression vector. Cell lines were grown at 37 °C in a humidified atmosphere with 5% CO_2_. For transfection, cells were cultured to 70% confluence and transfected with plasmids using Lipofectamine™ 2000 (Invitrogen, Carlsbad, CA) according to the manufacturer’s recommendations.

### Cell proliferation assay

For growth curve experiments, different experimental groups of A549 and H1299 cells were plated in 96-well plates at 1 × 10^4^ cells per well and incubated for 48 h after transfection. Optical density (OD) was measured using water-soluble tetrazolium salt assay and microplate computer software (Bio-Rad Laboratories, Hercules, CA) according to Cell Counting Kit-8 (CCK-8) assay kit instructions (Dojindo Laboratories, Japan). Absorbance at 450 nm was read on a microplate reader (168–1000 Model 680, Bio-Rad), and proliferation curves were plotted. Cell proliferation was measured using the ratio of OD of transfected cells in each group to ODs of blank control cells in each group. Data were expressed as percents of control.

### Colony formation assay

To measure colony-forming activity, three groups of A549 and H1299 cells were counted and seeded into 12-well plates (100 cells/well). Culture medium was replaced every 3 days. Twelve days after seeding, colonies containing more than 50 cells were counted.

### Construction of 3′-UTR-luciferase plasmid and reporter assays

The 3′-UTR of the SphK2 fragment was PCR-amplified from human genomic DNA and inserted into the pmiR-GLO control vector (Promega, Madison, WI) at the *Xho*I and *Xba*I sites 3′ to the luciferase gene. Primer sequences used for PCR amplification were as follows: forward 5′-AUGGGACCAGACGUGAUGCUGGA-3′, reverse 5′-GUUGUUUUAGUGACUACGACCU-3′. The 3′-UTR of SphK2 was confirmed with sequencing and named pmiR-GLO-WT. Site-directed mutagenesis of the miR-338-3p target site in the SphK2 3′-UTR (pmiR-GLO-mut) was carried out using a Quikchange site-directed mutagenesis kit (Promega, Madison, WI), with pmiR-GLO-WT as the template. For the luciferase reporter assay, A549 and H1299 cells were cultured in 96-well plates. Then, using Lipofectamine™ 2000 (Invitrogen, Carlsbad, CA), they were each cotransfected with wildtype or mutant reporter plasmid (100 nM) and microRNA (100 nM). At 48 h after transfection, luciferase activity was measured using the dual-luciferase assay system (Promega, Madison, WI).

### Apoptosis measurement using flow cytometry

A549 and H1299 cells were harvested 48 h after transfection and cell concentration was adjusted to 1 × 10^6^ cells. Annexin V-FITC/PI Apoptosis Detetion Kit Ι (BestBio, Shanghai, China) was used to measure Annexin V. Results were obtained using FACScan Flow Cytometer (BD Biosciences, San Jose, CA). Tests were repeated in triplicate. Data were analyzed with Cell Quest software.

### Animals and subcutaneous tumor growth

Male athymic nude mice (6–8 weeks-of-age) were obtained from the Animal Experimental Center of ZhengZhou University and were acclimated for 2 weeks. This study was conducted in accordance with the recommendations of the Guide for the Care and Use of Laboratory Animals of ZhengZhou University. The protocol was approved by the Committee on the Ethics of Animal Experiments of ZhengZhou University.

For in vivo tumorigenesis assays, all surgeries were performed under sodium pentobarbital anesthesia, and all efforts were made to minimize suffering. NSCLC A549 and H1299 cell line stably expressing luciferase infected with lentivirus packaged with lentiviral vectors LV6-miR-338-3p or LV6 empty vector (GenePharma, Shanghai, China), and cells were collected and injected into the flanks of nude mice. Thirty minutes after injection, luciferase substrate was added (150 mg/kg) and luciferase activity was measured every 5 days using the same protocol. Live tumor images were measured every 5 days for 3 weeks and monitored with bioluminescent imaging (PerkinElmer, Fremont, CA).

### Statistical analysis

Statistical analysis was performed using SPSS software version 16.0. All data are presented as mean ± SD where applicable. Differences were analyzed with the Student’s *t* test. Differences were considered significant when *p* < 0.05.

## Results

### miR-338-3p expression is significantly reduced and SphK2 expression is significantly increased in NSCLC tissues

**Fig. 1:**
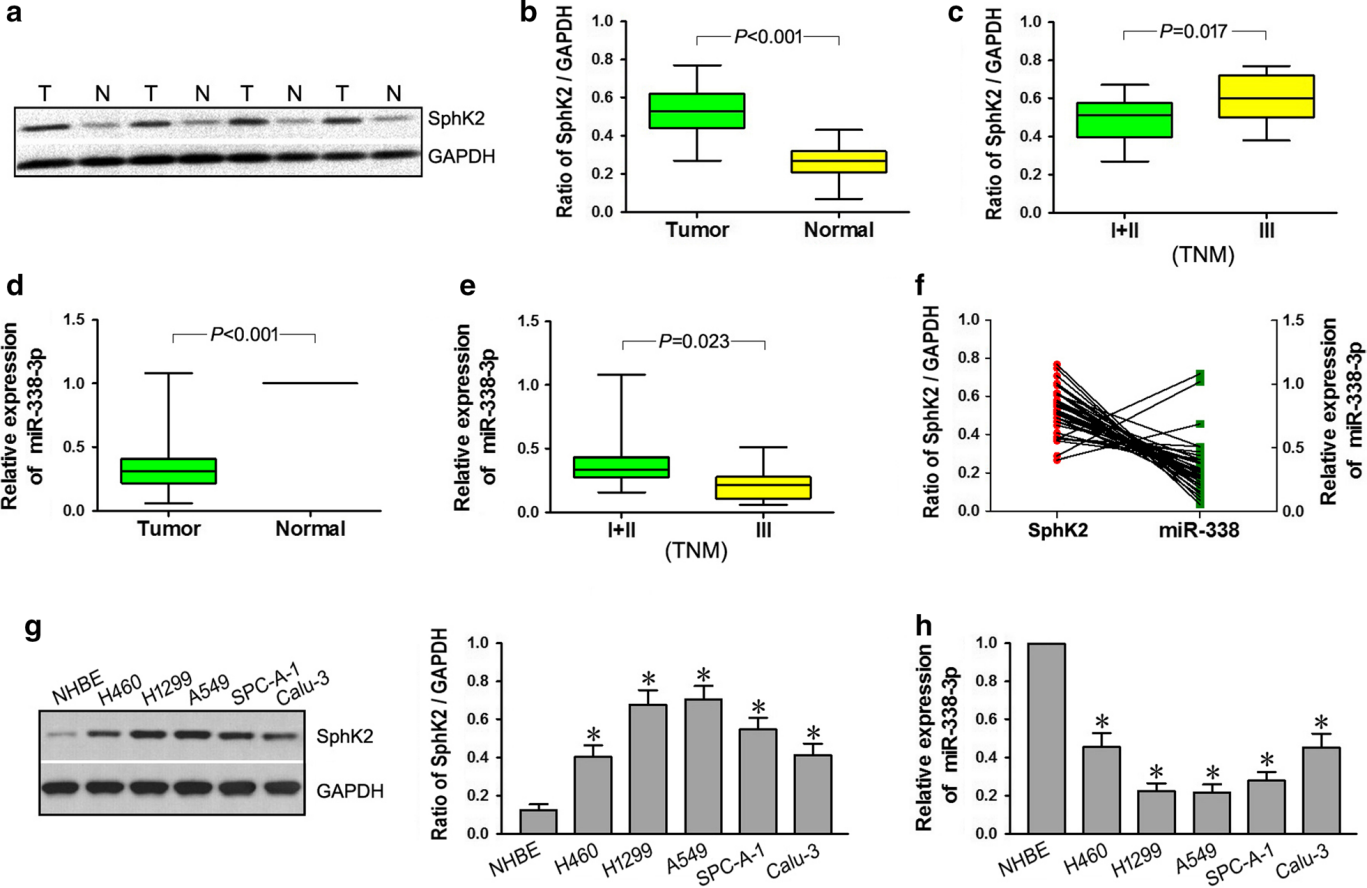
Expression of miR-338-3p and SphK2 in NSCLC. Relative expression of miR-338-3p was measured by qRT-PCR assay. U6 snRNA served as an endogenous control for normalization. SphK2 protein expression was measured using Western blot; GAPDH was an endogenous reference. Ratio of SphK2/GAPDH was equal to the protein expression level of SphK2 divided by the GAPDH protein expression level. **a** Western blot for SphK2 expression in four pairs of NSCLC tissues (T) and corresponding distant normal tissues (N). **b** Ratio of SphK2/GAPDH differed significantly between cancer tissues and corresponding distant normal tissues (*p < 0.05). **c** Ratio of SphK2/GAPDH in I + II stage cancer tissues and III stage cancer tissues of NSCLC with different TNM stages (*p < 0.05). **d** miR-338-3p expression differed significantly between cancer tissues and corresponding distant normal tissues (*p < 0.05). **e** miR-338-3p expression in I + II stage cancer tissues and III stage cancer tissues of NSCLC with different TNM stages (*p < 0.05). **f** Expression of SphK2 and miR-338-3p in NSCLC was negatively correlated. **g** Protein expression of SphK2 differed significantly between NHBE cell line and human lung cancer cell lines. **h** Relative expression of miR-338-3p differed significantly between NHBE cell line and human lung cancer cell lines

Analysis of patient data indicated that SphK2 expression was significantly correlated with NSCLC TNM stage (*p* = 0.017), and expression of miR-338-3p was also significantly correlated with NSCLC TNM stage (*p* = 0.023). Western blot confirmed that, compared to the distant normal tissues, SphK2 protein expression in NSCLC cancer tissues were higher (Fig. 1a). The results of half quantitative analysis for protein by Western blot indicated that the relative expression of SphK2 in NSCLC tumor tissues was much higher than in normal tissues (*p* < 0.001; Fig. 1b), and the relative expression of SphK2 in NSCLC I + II stage tumor tissues was lower than in NSCLC III stage tumor tissues (*p* = 0.017; Fig. 1c). qRT-PCR results showed that the relative expression of miR-338-3p in NSCLC tumor tissues was much lower than in normal tissues (*p* < 0.001; Fig. 1d), and the relative expression of miR-338-3p in I + II stage tumor tissues was higher than in III stage tumor tissues of NSCLC (*p* = 0.023; Fig. 1e). Thus, relative expression of SphK2 and miR-338-3p in NSCLC was negatively correlated (Fig. 1f). Results of western blot indicated that protein expression level of SphK2 in human lung cancer cell lines H460, H1299, A549, SPC-A-1, Calu-3 was much higher than that in NHBE, and more obvious in H1299 and A549 cell lines (Fig. 1g). qRT-PCR of the relative expression of miR-338-3p showed the opposite results, in human lung cancer cell lines H460, H1299, A549, SPC-A-1 and Calu-3, it was much lower than that in NHBE, and also more obvious in H1299 and A549 cell lines (Fig. 1h). Thus, the lung cancer cell lines used in the following experiments are H1299 and A549.

### miR-338-3p suppresses SphK2 expression by directly targeting the SphK2 3′-UTR

**Fig. 2:**
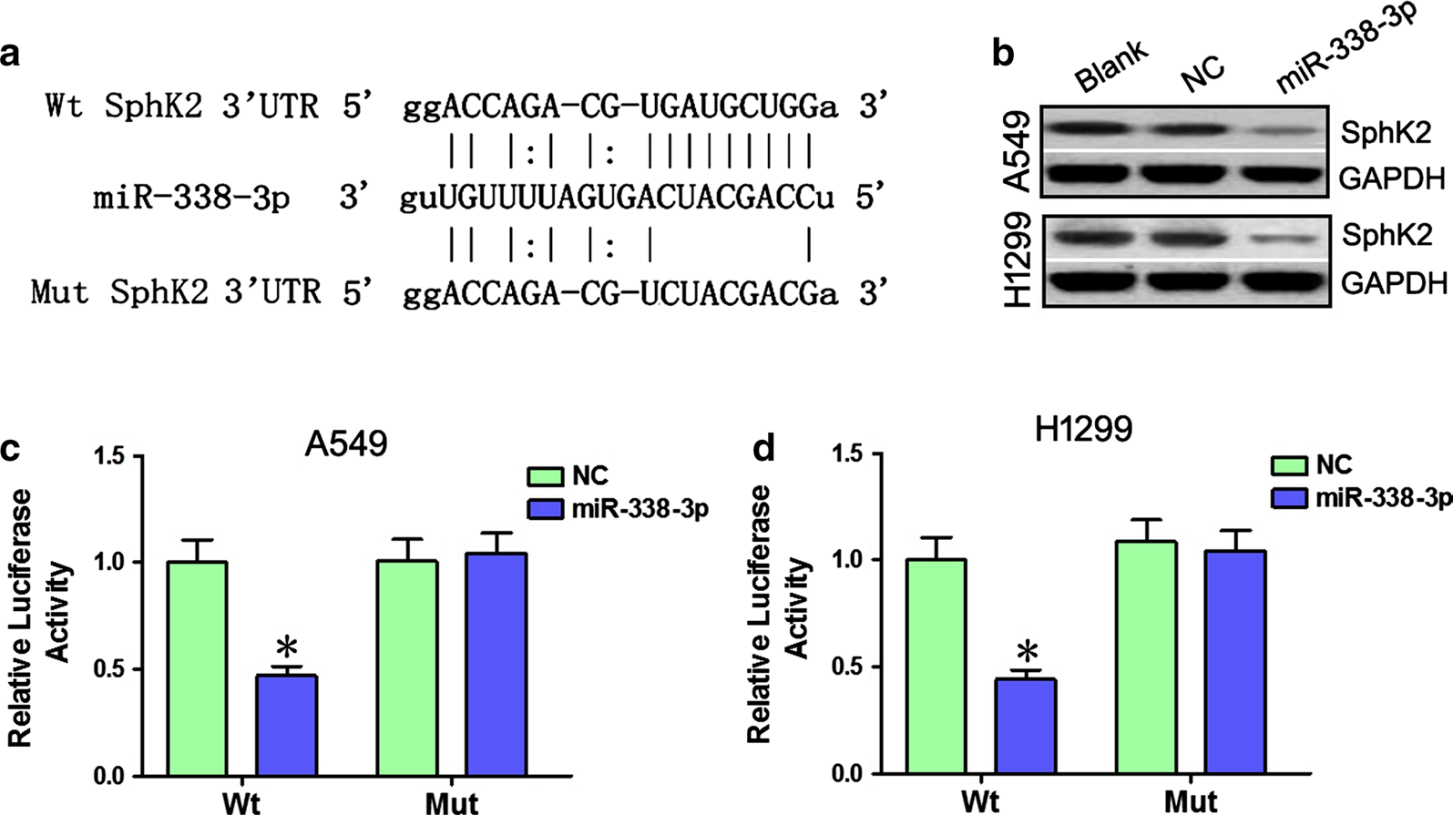
miR-338-3p directly targets SphK2. SphK2 protein expressions were measured by Western blot; Blank, untransfected cells; NC, cells transfected with negative control; GAPDH served as an endogenous reference. Luciferase reporter assays were used to assess the effect of miR-338-3p on SphK2; NC, cells transfected with negative control. **a** The putative wild-type SphK2 3′-UTR binding sequences in miR-338-3p, and the mutation sequence of SphK2 3′-UTR was not matched with the miR-338-3p. **b** Western blot quantification of SphK2 expression in transfected cells. GAPDH was an endogenous reference. miR-338-3p significantly downregulated expression of SphK2 in A549 and H1299 cells. **c** Luciferase reporter vectors that contained wild-type (or mutant-type) 3′-UTR segments of SphK2 were constructed and co-transfected into A549 cells with NC or miR-338-3p. Co-transfection of miR-338-3p significantly decreased luciferase activity of the reporter containing pmirGLO-wt 3′-UTR (*p < 0.05), but did not decrease that of the pmirGLO-mut 3′-UTR reporter. **d** Luciferase reporter vectors that contained wild-type (or mutant-type) 3′-UTR segments of SphK2 were constructed and co-transfected into H1299 cells together with NC or miR-338-3p. Co-transfection of miR-338-3p significantly decreased luciferase activity of the reporter containing pmirGLO-wt 3′-UTR (*p < 0.05), but did not decrease that of the pmirGLO-mut 3′-UTR reporter

Bioinformatic analysis using TargetScan and miRanda indicated that the 3′-UTR of SphK2 contains a predicted binding site for miR-338-3p (Fig. 2a). Western blot and luciferase reporter assays were used to determine whether SphK2 is regulated by miR-338-3p. Western blot indicated that SphK2 expression was downregulated in A549 and H1299 cells after transfection with miR-338-3p (Fig. 2b). A dual-luciferase reporter system with luciferase reporter vectors containing either the wild-type or the mutant 3′-UTR of SphK2 verified whether SphK2 is a direct target of miR-338-3p. Co-transfection of miR-338-3p significantly decreased luciferase activity of the reporter containing wild-type 3′-UTR, but did not decrease that of the mutant 3′-UTR reporter (*p < 0.05) in A549 cells (Fig. 2c). Also, co-transfection of miR-338-3p significantly decreased luciferase activity of the reporter containing the wild-type 3′-UTR reporter in H1299 cells (*p < 0.05; Fig. 2d). Thus, SphK2 is a direct functional target of miR-338-3p, which negatively regulates SphK2 expression by directly binding to its putative binding site in the 3′-UTR sequence.

### Overexpression of miR-338-3p suppresses proliferation of A549 and H1299 cells

**Fig. 3:**
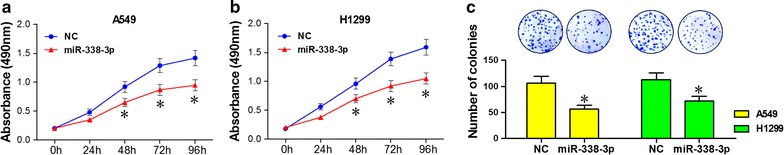
miR-338-3p reduces NSCLC cell viability. CCK-8 assay and colony formation assay were used to measure the effect of miR-338-3p on NSCLC viability. Data are presented as means of three repeated experiments. NC means cells transfected with negative control. **a** CCK-8 assay confirmed that miR-338-3p inhibited A549 cell growth. **b** CCK-8 assay confirmed that miR-338-3p inhibited growth of H1299 cells. **c** Colony formation confirmed that in A549 and H1299 cells, miR-338-3p reduced colonies compared with NCs (*p < 0.05)

CCK-8 assays and colony formation assays were performed in NSCLC cells to evaluate the effect of miR-338-3p on cell proliferation. Data show that compared with NCs cell viability of the miR-338-3p group decreased from 24 h onwards in A549 (Fig. 3a) and H1299 (Fig. 3b) cells. Also, colony formation in the miR-338-3p group was much lower than that of NCs in A549 and H1299 cells (Fig. 3c). Thus, miR-338-3p suppresses viability of NSCLC A549 and H1299 cells.

### miR-338-3p induces apoptosis of A549 and H1299 NSCLC

**Fig. 4:**
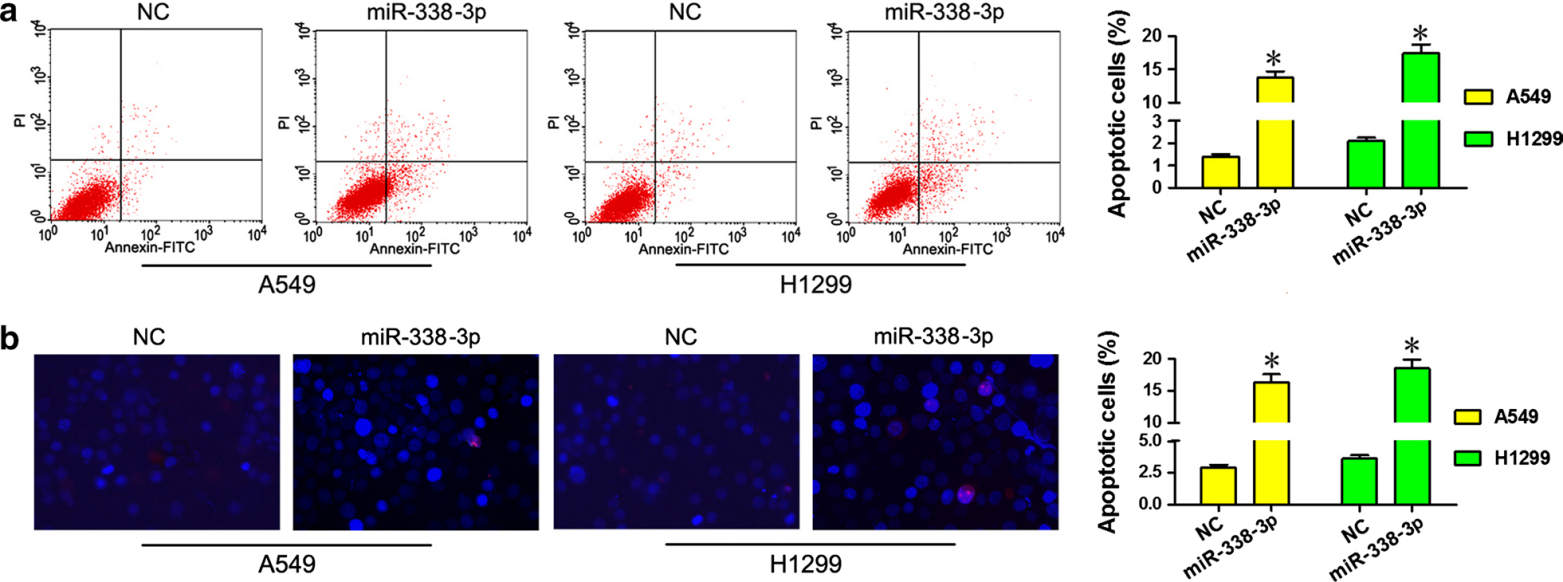
miR-338-3p promotes apoptosis. *FITC* fluorescein isothiocyanate, *PI* propidium iodide, *NC* cells transfected with negative control. **a** At 48 h after transfection with miR-338-3p or NC, A549 cells or H1299 cells was collected for analysis of apoptosis. Flow cytometry was used to measure apoptosis after 2 days in A549 or H1299 cells transfected with miR-338-3p or NC. **b** Percent of NSCLC apoptosis was calculated using TUNEL staining. Apoptosis of A549 or H1299 cells transfected with miR-338-3p or NC measured after 2 days

Flow cytometry assay indicate that, after A549 and H1299 cells were transfected with miR-338-3p for 48 h, Annexin-FITC positive A549 cells were significantly increased compared with NCs, and results of H1299 cells were similar (Fig. 4a). TUNEL staining to measure NSCLC apoptosis indicated that apoptosis increased compared to NCs (Fig. 4b).

### miR-338-3p inhibits NSCLC cells A549 and H1299 growth in vivo

**Fig. 5:**
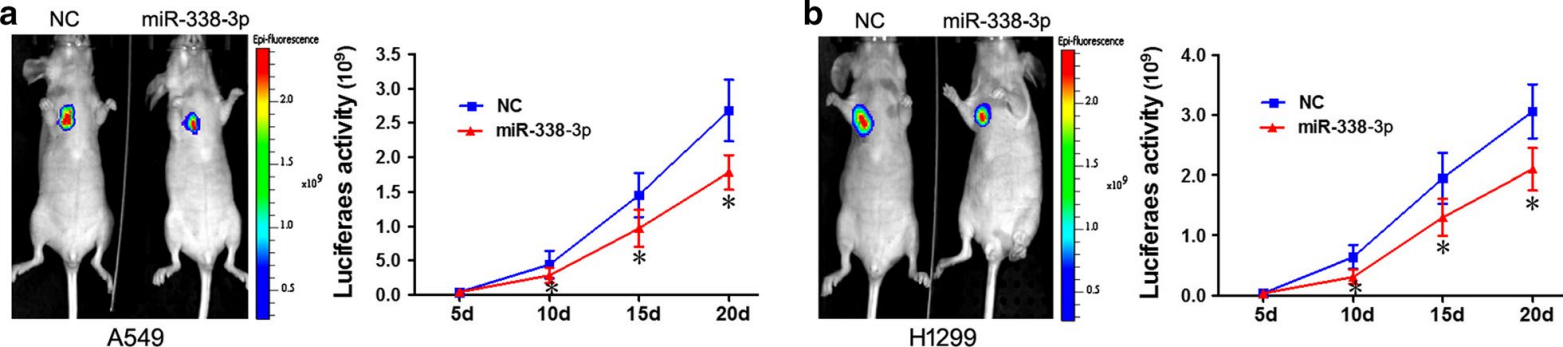
miR-338-3p inhibits subcutaneous tumor growth. NSCLC A549 and H1299 cell line stably expressing luciferase infected by lentivirus packaged with vectors LV6-miR-338-3p or LV6 empty vector as described in “Methods”. Live images of tumors were measured every 3 days for 4 weeks and monitored with bioluminescent imaging. Thirty minutes after cell injection, luciferase substrate was added (150 mg/kg) and luciferase activity was measured every 5 days using the same protocol. Data are mean ± SD (*p < 0.05, *t* test). **a** Representative images of tumors on indicated days (*left panel*) and tumor growth curve in mice are shown (*right panel*) for A549 cells. **b** Representative images of tumors on indicated days (*left panel*) and tumor growth curve in mice (*right panel*) for H1299 cells

Studies with human NSCLC xenografts in nude mice indicated that bioluminescence in the miR-338-3p group less than in NCs and tumor growth curves for mice in the miR-338-3p group was less than NCs (Fig. 5a). H1299 cells were similar (Fig. 5b). Thus, miR-338-3p inhibited tumorigenicity of NSCLC A549 and H1299 cells in a nude mouse xenograft model.

### Inhibitory effect of miR-338-3p on NSCLC A549 and H1299 cells is mediated by down-regulating SphK2

**Fig. 6:**
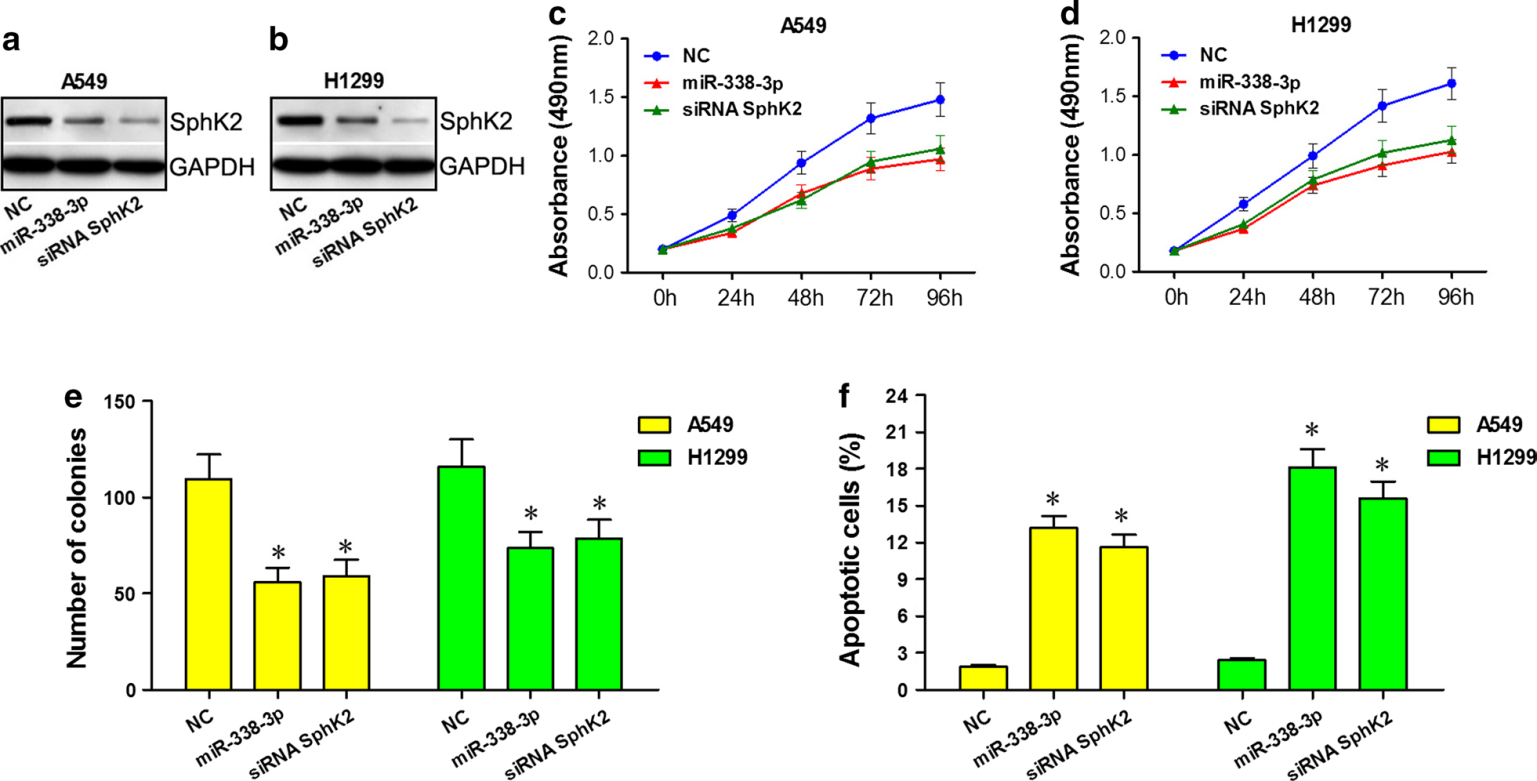
Inhibitory effect of miR-338-3p on NSCLC is mediated by downregulating SphK2. GAPDH was an endogenous reference; NC, cells transfected with negative control. **a** SphK2 protein was measured using Western blot which showed that transfection of SphK2-siRNAs and miR-338-3p inhibited expression of SphK2 in A549 cells, respectively. **b** SphK2 protein (Western blot) indicated that transfection of SphK2-siRNAs and miR-338-3p inhibited expression of SphK2 in H1299 cells, respectively. **c** CCK-8 assay showed that SphK2-siRNA and miR-338-3p inhibited A549 cell proliferation. **d** CCK-8 array showed that SphK2-siRNA and miR-338-3p inhibited H1299 cell proliferation. **e** Colony formation assay showed that SphK2-siRNA and miR-338-3p reduced A549 and H1299 cell colony growth. **f** Flow cytometry showed that SphK2-siRNA and miR-338-3p induced A549 and H1299 apoptosis

Western blot indicated that transfection of SphK2-siRNA and miR-338-3p inhibited expression of SphK2, respectively (Fig. 6a, b). CCK-8 assay showed that SphK2-siRNA inhibited proliferation of A549 and H1299 cells compared to NCs, and this was similar to cells transfected with miR-338-3p (Fig. 6c, d). A colony formation assay indicated that SphK2-siRNA reduced colonies of A549 and H1299 cells compared to NCs, and these reductions were similar to cells transfected with miR-338-3p (Fig. 6e). Flow cytometry confirmed that SphK2-siRNA induced apoptosis of A549 and H1299 cells compared to NCs, and activation was similar to cells transfected with miR-338-3p (Fig. 6f). Thus, miR-338-3p inhibited NSCLC biological effects by down-regulating SphK2.

### Restoration of SphK2 rescues tumor suppression by miR-338-3p

**Fig. 7:**
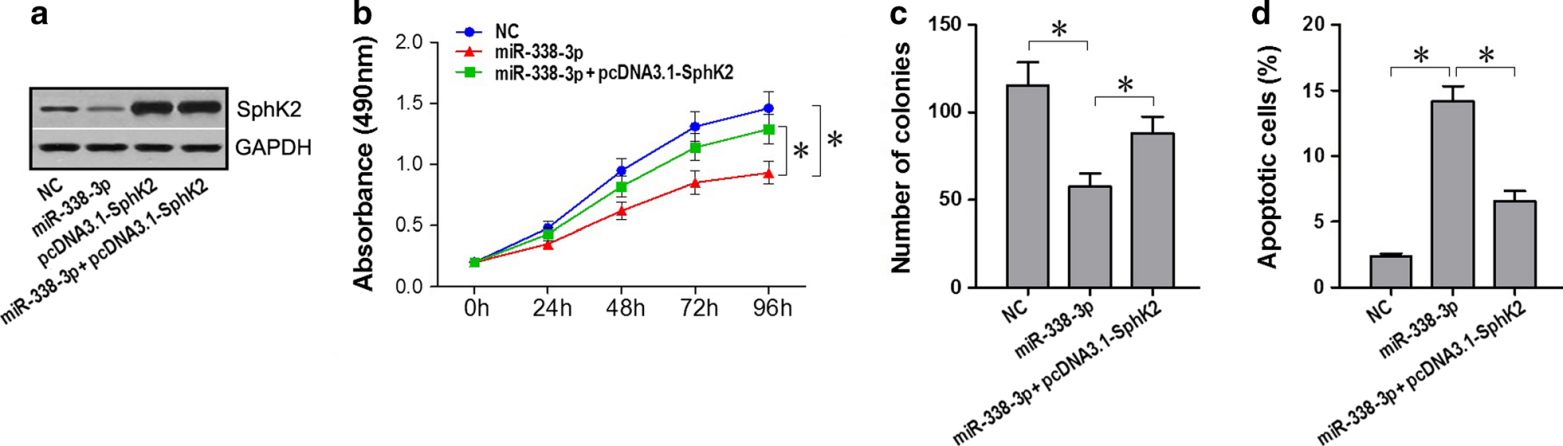
Over-expression of SphK2 lacking the 3′-UTR restores the effects of miR-338-3p on NSCLC cell proliferation and apoptosis. NSCLC cell line was co-transfected with miR-338-3p and pcDNA3.1-SphK2 without the 3′-UTR. GAPDH was an endogenous reference; NC, cells transfected with negative control. **a** SphK2 expression was measured using western blots for each group. **b**–**d** Cell proliferation by CCK-8 assays and by colony formation assays, cell apoptosis by flow cytometry assays (*p < 0.05)

To investigate whether the effects of miR-338-3p on the cell proliferation and apoptosis of NSCLC cells was mediated by SphK2 repression, we overexpressed SphK2 lacking the 3′-UTR in NSCLC cell lines and co-transfected with miR-338-3p. Results of western blot showed that expression level of SphK2 protein was downregulated in A549 cells after transfected with miR-338-3p, and overexpressed both in cells transfected with pcDNA3.1-SphK2 (without the 3′-UTR) alone and co-transfected with miR-338-3p. In addition, expression level of SphK2 protein showed no significant difference between the later two groups (Fig. 7a). Results of CCK-8 assay and colony formation assay showed that the proliferation inhibitory effects of miR-338-3p on A549 cells were partly restored by pcDNA3.1-SphK2 lacking the 3′-UTR (Fig. 7b, c), and the apoptosis promoted effects of miR-338-3p were also partly restored (Fig. 7d). These results indicated that the effects of miR-338-3p on NSCLC cell proliferation and apoptosis were restored by SphK2 lacking the 3′-UTR, suggesting that miR-338-3p suppress NSCLC cell proliferation and induce apoptosis by targeting the 3′-UTR of SphK2.

## Discussion

miRNAs are small, noncoding RNAs, 21–25 nucleotides in length, which are master gene mediators that form the miRNA-induced silencing complex (miRISC) and lead to mRNA instability or degradation [24]. Aberrant miRNA expression occurs in many biological processes such as cell proliferation, the cell cycle, apoptosis, invasion, and migration. Depending on the cellular function of certain miRNA targets, miRNAs can behave as oncogenes or tumor suppressor genes.

The apoptosis-associated tyrosine kinase (*AATK*) gene is located on chromosome 17 (17q25.3) [25]. Studies indicate that the role of *AATK* in anti-tumorigenesis and aberrant Aatk expression depends on methylation in the CpG island promoter of Aatk [26, 27]. miR-338-3p suppresses the translation of a select group of cellular mRNA whose protein products are negative regulators of neurite growth. Previously, miR-338-3p was shown to act as a tumor suppressor in some cancers [28, 29].

Previous studies indicate that miR-338-3p is downregulated in colorectal and hepatocellular carcinomas and gastric cancer [30–32]. However, the expression pattern of miR-338-3p in lung cancer, particularly in NSCLC, is unreported. Data from miRNA arrays indicate that miR-338-3p is downregulated in NSCLC tissues [33, 34] and miR-338-3p may exert a tumor suppressor role in NSCLC. Using various approaches, we observed that overexpression of miR-338-3p suppressed NSCLC A549 and H1299 cell proliferation and induced apoptosis in vitro and in vivo.

Identification of targets of miR-338-3p in NSCLC is necessary for understanding the underlying regulatory mechanisms so we used bioinformatics for target gene prediction. Considering overlap of the genes identified by TargetScan, miRBase targets and PicTarget, SphK2 was selected to be a potential target for validation. Using luciferase reporter assays, Western blot, and qRT-PCR assays we verified that miR-338-3p directly targets SphK2 by interacting with the first binding site in the 3′-UTR.

## Conclusions

We found that miR-338-3p was downregulated in NSCLC tissues, and was significantly correlated with NSCLC pathological stage. miR-338-3p overexpression suppressed NSCLC cell proliferation and induced apoptosis as well as directly targeted SphK2 and inhibited effect of miR-338-3p on NSCLC A549 and H1299 cells by down-regulating SphK2. And miR-338-3p inhibited NSCLC cell growth in vivo. However, more studies are needed with more clinical samples to determine the clinical significance and prognostic value.

## Abbreviations

NSCLC: non-small-cell lung cancer
miR-338-3p: miRNA-338-3p
SphK2: sphingosine kinase 2
CCK-8: cell counting kit 8
3′-UTR: 3′-untranslated region
AATK: apoptosis-associated tyrosine kinase
FBS: fetal bovine serum
DMEM: Dulbecco’s minimum essential medium
NC: negative control
